# Periarticular Injection with Bupivacaine for Postoperative Pain Control in Total Knee Replacement: A Prospective Randomized Double-Blind Controlled Trial

**DOI:** 10.1155/2012/107309

**Published:** 2012-10-22

**Authors:** Varah Yuenyongviwat, Chaturong Pornrattanamaneewong, Thitima Chinachoti, Keerati Chareancholvanich

**Affiliations:** ^1^Department of Orthopaedic Surgery, Faculty of Medicine, Siriraj Hospital, Mahidol University, Bangkok 10700, Thailand; ^2^Department of Anesthesiology, Faculty of Medicine, Siriraj Hospital, Mahidol University, Bangkok 10700, Thailand

## Abstract

*Background*. Local periarticular injection with bupivacaine alone in TKA has not been studied. Thus, we aimed to examine the effectiveness of local periarticular injection with bupivacaine for post-operative pain control in TKA. *Method*. Sixty patients undergoing TKA by a single surgeon were randomly assigned into two groups in a double-blind, placebo-controlled study. In the injection group, patients received periarticular injections with 0.25% bupivacaine before wound closure; in the control group, patients received a 0.9% normal saline injection. Both groups received the same anesthetic procedure, post-operative pain control, and rehabilitation protocol. *Results*. There was a significant reduction in post-operative morphine consumption in the first six hours after the operation (mean 0.9 mg and 2.43 mg, *P* = 0.01), but there was no significant difference in post-operative morphine consumption between six hours and ninety-six hours after the operation, visual analogue scale (VAS) score, morphine side effects during the first 96 hours, length of hospital stay, or complications from morphine consumption. *Conclusion*. Local periarticular injection with bupivacaine alone before wound closer was shown to be an effective method to improve pain control after TKA with a few complications and ease of use.

## 1. Introduction

Total knee arthroplasty is a procedure that can improve quality of life, and it is performed in increasing numbers every year because of the increase in the elderly population due to improved medical technology [1]. However, the problem is that patients avoid this operation because of post-operative pain, which affects patient satisfaction and delays recovery and rehabilitation [2].

 There are many modalities to improve operative pain control, such as femoral nerve blocks [3], epidural anesthesia [4, 5], and periarticular injections [6–11]. These modalities have been shown to reduce post-operative pain and increase patient satisfaction. 

 Periarticular injection was reported to have good efficacy in controlling pain, cost effectiveness, a few side effects, and ease of use [6–8]. This method can be used by every surgeon without further training, unlike techniques like epidural anesthesia or femoral nerve blocks, which require experience and further training.

 Many studies about periarticular injection have reported good results from various medications and combinations, such as ropivacaine, ketorolac, and epinephrine [6, 7]; bupivacaine, morphine sulfate, epinephrine, methyprednisolone acetate, cefuroxime, and normal saline [8–10]; ropivacaine, ketorolac, epimorphine, and epinephrine [11]. All of these combinations were reported with good efficacy to control pain with a few complications. However, there have not been studies that show which of the medications in these combinations has the most important role in improving post-operative pain control. 

The efficacy of local injection with bupivacaine alone was reported with good results in lumbar discectomy and lumbar laminectomy. It was found to prolong the post-operative narcotic-free duration and to decrease the amount of post-operative parenteral analgesia [12, 13]. Our hospital protocol for total knee replacement also uses periarticular injection with Bupivacaine alone.

 The primary aim of this prospective, randomized, double-blind, controlled trial was to evaluate the efficacy of periarticular injection with bupivacaine for reducing patient reported post-operative pain as assessed by morphine consumption via PCA.

## 2. Materials and Methods

### 2.1. Participants

 Sixty primary osteoarthritis patients undergoing total knee replacement at Siriraj Hospital were included in this study. Inclusion criteria were patient age between fifty to eighty years, BMI between twenty-five to thirty-five, and having a full understanding of the question in this trial. Exclusion criteria were patients with a history of allergy to the medication used in this trial, a renal problem (blood creatinine more than 1.5 mg/dL or creatinine clearance less than 60), abnormal liver function, previous surgery on the knee undergoing total knee replacement, and those who could not receive spinal anesthesia. All included patients signed an informed consent form, and the methods of this trial were approved by the ethics committee (Siriraj Institutional Review Board, Faculty of Medicine, Siriraj Hospital, Mahidol University.) The procedures in this study were in accordance with the Declaration of Helsinki on ethical principles for medical research involving human subjects.

### 2.2. Study Protocol

 This was a parallel, prospective, randomized, double-blind, controlled trial. All patients received spinal anesthesia with 3 mL of 0.5% heavy bupivacaine and morphine (0.3 mg) by an experienced anesthetist. Cefazolin (1 g) was used for prophylaxis against surgical infection thirty minutes prior to starting the operation. Clindamycin (600 mg) was used in patients with a penicillin or cephalosporin allergy history. Ondansetron (8 mg) was injected at the start of the operation, and an additional 8 mg was given 12 hours later. Patients who were anxious during the operation received midazolam (1-2 mg) or propofol (50–100 mg/hr).

 All of the operations were performed by a single surgeon. A tourniquet was used with 350 mmHg of pressure while performing the surgery from the start of incision until the operation was finished. A standard minimedial parapatellar approach was used, and a redivac drain was placed in all patients. A posterior stabilized total knee prosthesis (Zimmer Nexgen Legacy: LPS-flex) with a cementing technique was used in every operation.

### 2.3. Randomization and Blinding

 Patients were randomized to one of two treatment groups (simple randomization by sealed envelope) in the operating room before the initial incision by a study coordinator who prepared the medication for periarticular injection and was not involved in the operation or patient care. The physicians, patients, and nurses who collected postoperative patients data were blinded to treatment group allocation.

### 2.4. Interventions

 The first group was patients receiving a periarticular injection with 20 mL of 0.25% bupivacaine before wound closure. The second group was patients receiving a periarticular injection with 20 mL of 0.9% normal saline before wound closure. Periarticular injection was performed with a 21-gauge catheterfrom inside wound at the extensor mechanism (3 mL), capsule (5 mL), pes anserinus (1 mL), iliotibial band (1 mL), collateral ligament (2 mL), and subcutaneous tissue (8 mL). 

 For post-operative pain control, patients received an intramuscular injection of diclofenac (50 mg) after the operation. Patients also took acetaminophen (1 g) every 6 hours and meloxicam (7.5 mg) every 12 hours. Patient-controlled analgesia (PCA) was used in all patients for 96 hours postoperatively. 

The PCA administration was morphine 1 mg IV bolused with 15-minute lock-out interval.

 The day after the operation, the urine catheter was removed, and range of motion and isometric exercises were started. Patients were encouraged to ambulate with a walker as tolerated.

## 3. Outcomes

### 3.1. Primary and Secondary Outcome Measures

 The primary outcome of this study is post-operative morphine consumption via PCA that was used to evaluate post-operative pain control, visual analog scale scores were record at rest in paper by trained nurses every 3 hours in 0–24 hours after operation then every 6 hours until 96 hours after operation.

The VAS consists of a 10 cm line where 0 indicates no pain and 10 representing the worst imaginable pain. Complications from morphine consumption such as nausea, pruritis, urinary retention, and constipation were also recorded. Wound was inspected for any complication at two weeks postoperatively.

### 3.2. Statistical Analysis

#### 3.2.1. Sample Size

Twenty-eight participants per study group were required to achieve a 90% chance of detecting a significant difference between groups (1.5 dose equivalent on mean morphine consumption in early postoperative period) with a standard deviation of 3.4 dose equivalent. The effect size was estimated from a previous study [14].

#### 3.2.2. Statistical Methods

 The analyses were performed with SPSS software (version 11.5; SPSS, Chicago, Ill, USA). Kolmogorov-Smirnov test was used to test for a normal distribution. Operative time was evaluated using the Student's *t*-test.

Complications from morphine consumption were analyzed by Chi-square test.

Post-operative morphine consumption via PCA, visual analog scale scores, and duration of hospital stay were compared between groups using Mann-Whitney *U* test. Statistical significance was assumed if *P* < 0.05.

## 4. Results

### 4.1. Participant Flow

**Figure 1:**
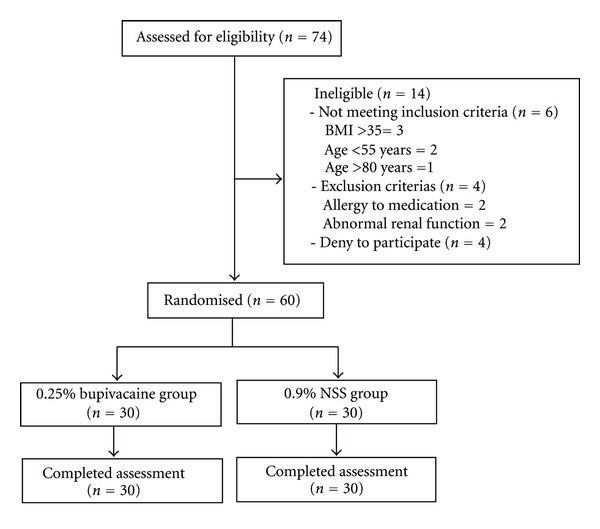
Participants flow.

 Patients were consecutive recruited between March 2010 and August 2010. Seventy-four patients were assess for eligibility. Of these, six did not meet the inclusion criteria, four met the exclusion criteria, and four refused to participate. Sixty patients were included in this study (Figure 1). 

Thirty patients received a periarticular injection with 0.25% bupavacaine, and the other thirty patients were assigned to a control group that received an injection of 0.9% normal saline. Both groups of patients had similar baseline characteristics.  Table 1 shows the demographic data of the patients in both groups. There was no significant difference in sex, side of operation, age, weight, height, BMI, and diabetes mellitus history (Table 1).

 The mean operative time in the bupivacaine group and control group was 82.50 (±16.80) minutes and 82.33 (±11.94) minutes, respectively. There was no significant difference between the groups (*P* = 0.96).

**Figure 2:**
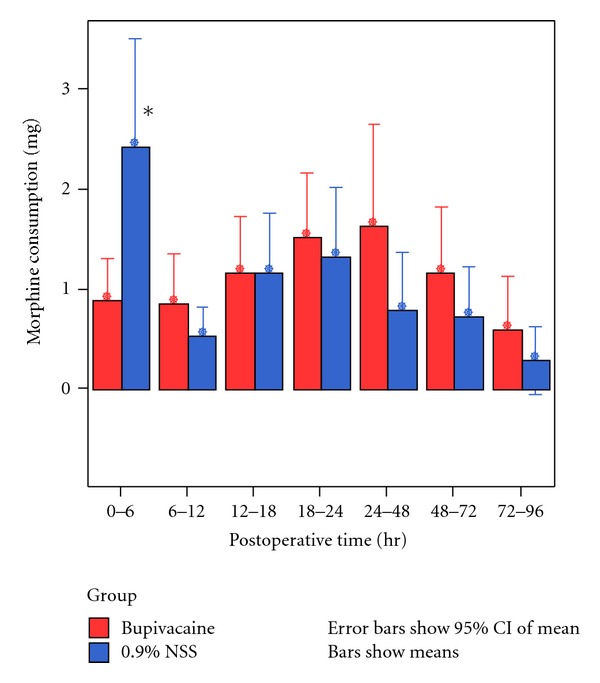
Post-operative morphine consumption at 96 hours after operation *P < 0.05.

 At 6 hours after the operation, the patients in the periarticular injection with 0.25% bupivacaine group had a significant reduction (*P* = 0.01) in PCA morphine consumption (mean 0.9 mg ± 1.13 mg) compared with the control group (mean 2.43 mg ± 2.91 mg). There was no significant difference between the groups at 6–12 hours after the operation (mean 0.87 ± 1.33 mg in the bupivacaine group and 0.53 ± 0.78 mg in the control group, *P* = 0.24), at 12–18 hours after the operation (mean 1.17 ± 1.53 mg in the bupivacaine group and 1.17 ± 1.60 mg in the control group,  *P* = 1.0), at 18–24 hours after the operation (1.53 ± 1.72 mg in the bupivacaine group and 1.33 ± 1.86 mg in the control group, *P* = 0.67), at 24–48 hours after the operation (1.63 ± 2.76 mg in the bupivacaine group versus 0.8 ± 1.56 mg in the control group, *P* = 1.56), at 48–72 hours after the operation (1.17 ± 1.78 mg in the bupivacaine group versus 0.73 ± 1.36 mg in the control group, *P* = 0.3) and at 72–96 hours after the operation (0.60 ± 1.45 mg in the bupivacaine group versus 0.30 ± 0.92 mg in the control group, *P* = 0.34) (Figure 2).

**Figure 3:**
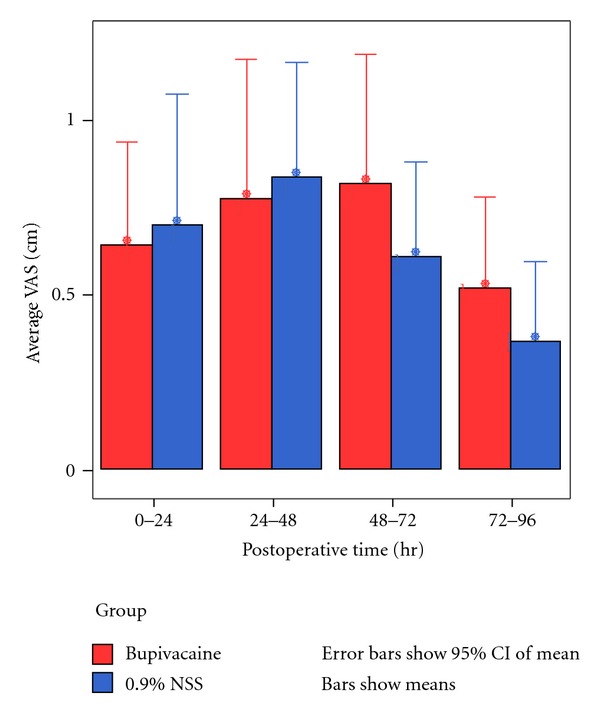
Postoperative mean visual analog scale score at 96 hours after operation.

 The mean visual analog scale score for operative pain was not significantly different in both groups at 0–96 hours after the operation (0–24 hours after the operation (mean 0.64 ± 0.79 in the bupivacaine group and 0.7 ± 1.0 in the control group, *P* = 0.94), 24–48 hours after the operation (0.77 ± 1.07 in the bupivacaine group and 0.83 ± 0.88 in the control group, *P* = 0.64), 48–72 hours after the operation (0.82 ± 0.98 in the bupivacaine group versus 0.60 ± 0.72 in the control group, *P* = 0.49), and 72–96 hours after the operation (0.52 ± 0.69 in the bupivacaine group versus 0.67 ± 0.61 in the control group, *P* = 0.45)) (Figure 3).

 Patients in the bupivacaine group had a mean length of hospital stay of 6.83 ± 0.59 days, while control patients had a mean length of stay of 6.87 ± 0.51 days. No difference was found in the length of hospital stay between the groups (*P* = 0.77). 

**Figure 4:**
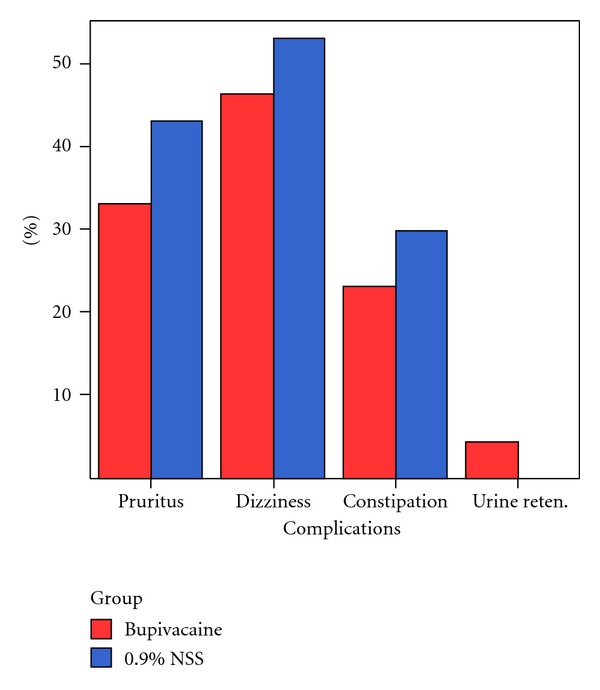
Morphine-related postoperative complications.

 Complications from morphine consumption, such as pruritus, nausea and vomiting, and urinary retention, were not statistically different between the groups, as shown in Figure 4. No wound complications were found in either group of patients at two-week post operative follow up. 

## 5. Discussion

 A multimodal technique was developed to improve post-operative pain control after total knee arthroplasty. Femoral nerve blocks [3] and epidural anesthesia [4, 5] have been reported to control pain with good efficacy. However, these procedures require a well-trained physician, and there are some complications that could result from these procedures. Sharma et al. reported the rate of femoral neuropathy/neuritis after femoral nerve block has been estimated to be approximately 0.59% [15]. 

 The intraoperative periarticular injection technique for reduction of post-operative pain control following total knee arthroplasty has been reported and has demonstrated good results. 

Vendittoli et al. reported that perioperative periarticular infiltration with ropivacaine, ketorolac, adrenaline, and injection with ropivacaine (150 mg) on the first after the operative day showed a reduction in narcotic requirements at 48 hours after the operation with minimal side effects to patients when compared to the control group [7].

Parvataneni et al. studied the efficacy of local intraoperative injection with a combination of 0.5% bupivacaine, morphine sulfate, epinephrine, methylprednisolone acetate, cefuroxime, and normal saline in total hip and total knee replacement patients. This study demonstrated a reduction in the pain score at 72 hours after the operation, a decreased hospital stay, and increased satisfaction scores when compared with the control group [8].

Busch et al. reported a decreased consumption of patient-controlled analgesia (PCA) at 12 hours after the operation and a lower pain score in total knee replacement patients who received a perioperative, periarticular injection with ropivacaine, ketorolac, epimorphine, normal saline, and epinephrine compared with patients who did not receive an injection [11].

In our study, we collected data from 0–96 hours that was the time when the patients had PCA. Data from our study are in agreement with previous reports on the efficacy of periarticular injection. However, our study objective was to evaluate the efficacy of bupivacaine alone in reducing post-operative pain when used for perioperative, periarticular injection. Our study demonstrated that bupivacaine injection alone, without other drugs, significantly reduced morphine consumption at 6 hours after the operation. However, visual analog scale scores for pain were not different between the control and bupivacaine-injection groups. This may be because morphine that the control group patients received which was more than the other groups via PCA obscured VAS scores. So, the result confirmed that the patients used PCA machine properly when they started to feel pain. Other reason that this study lacks having an effect on the VAS scores may be the issue of dosing that the previous study used higher dose of anesthetic agent than our study.

We did not observe complications from a periarticular bupivacaine injection in our study, which is consistent with previous periarticular injection studies. Thus, our study adds to evidence that periarticular injections are safe when used in knee replacement procedures, in which post-operative infection and wound complications are the most prominent complications.

## 6. Conclusions

In conclusion, intraoperative periarticular injection with 0.25% bupivacaine alone is effective in reducing post-operative morphine consumption with few complications. Furthermore, this treatment is as easy to use as periarticular injections with cocktail formulas demonstrated in previous studies.

## Figures and Tables

**Table 1:** Patient baseline demographic data.

Treatment group	Bupivacaine injection	0.9% NSS injection	*P*
Sex			
Male	2	2	1.0
Female	28	28	
Side			
Right	17	10	0.07
Left	13	20	
Diabetes mellitus	9	8	0.77
Mean age ± SD	69.27 ± 6.89	70.43 ± 5.63	0.48
Mean weight (kg) ± SD	65.28 ± 10.38	63.83 ± 6.73	0.52
Mean height (cm) ± SD	154.35 ± 7.84	152.74 ± 7.14	0.41
BMI ± SD	27.10 ± 4	27.41 ± 2.8	0.73
